# Genome Sequence of *Cupriavidus metallidurans* Strain H1130, Isolated from an Invasive Human Infection

**DOI:** 10.1128/genomeA.01051-13

**Published:** 2013-12-12

**Authors:** Pieter Monsieurs, Ann Provoost, Kristel Mijnendonckx, Natalie Leys, Christiane Gaudreau, Rob Van Houdt

**Affiliations:** Unit of Microbiology, Belgian Nuclear Research Centre (SCK·CEN), Mol, Belgiuma; Microbiologie Médicale et Infectiologie, Centre Hospitalier de l’Université de Montréal (CHUM)-Hôpital Saint-Luc, Montréal, Québec, Canadab

## Abstract

*Cupriavidus metallidurans* H1130 was repeatedly isolated from different blood culture sets taken from a patient suffering from significant nosocomial septicemia. Here, we announce the H1130 genome sequence for use in comparative analyses and for exploring the adaptation and pathogenic potential of this bacterium.

## GENOME ANNOUNCEMENT

*Cupriavidus metallidurans* strains are frequently isolated from industrial sites linked to mining, metallurgic, and chemical industries (1–3), as well as from other anthropogenic environments not typified by metal contamination, such as spacecraft-related environments (4). *C. metallidurans* strains are Gram-negative, peritrichously flagellated bacterial rods with an oxidative metabolism (5) and are characterized by multiple metal resistances (1, 4, 6–8). Many of the metal resistance determinants are shared by all *C. metallidurans* strains, irrespective of the isolation type and location of a strain (6), and a substantial number of these determinants are carried by native megaplasmids like pMOL28 and pMOL30 of type strain CH34 (9). *C. metallidurans* strains do display substantial differences in their mobile gene pools (6), which include genomic islands (GIs), integrative and conjugative elements, transposons, and insertion sequence (IS) elements (10, 11).

*C. metallidurans* isolates are increasingly being recovered from medically relevant sources, such as patients with cystic fibrosis (12) and human cerebrospinal fluid (e.g., strain CCUG43015, deposited directly to the Culture Collection, University of Göteborg). Recently, the first case of invasive infection by *C. metallidurans* was reported (13). Four out of 5 blood culture sets taken over a period of 6 days from a patient with signs of sepsis were positive for a Gram-negative rod that was identified as *C. metallidurans* (GenBank accession no. GU230889). The genome of this isolate, designated H1130, was sequenced for comparative analyses and for exploring the adaptation and pathogenic potential of this bacterium.

Full-genome sequencing of *C. metallidurans* H1130 was performed by BaseClear (Leiden, The Netherlands) on the Illumina HiSeq 2000 platform using a paired-end sequencing library (50 nucleotides) (Illumina, Inc.), with an average insert size of 328 nucleotides (nt). The reads were filtered to remove low-quality reads, resulting in a total of 6,894,135 paired reads (7.03 Gbp). The genome of *C. metallidurans* H1130 was estimated to be 7,225,099 bp, which was sequenced with >98-fold genome coverage. *De novo* assembly of the paired reads was performed using two different genome assembly tools, Velvet (hash length, 37; minimal contig length, 500; minimal coverage cov_cutoff, 6) and ABySS (parameter k set to 45), resulting in 683 and 1,099 contigs, respectively. Both assemblies were integrated using the Minimus software, resulting in a total of 118 assembled contigs.

Three replicons were identified, namely, 1 chromosome, 1 secondary chromosome or chromid (presence is phylogenetically linked [14]), and 1 megaplasmid (based on megaplasmid extraction analysis; for the method, see Mijnendockx et al. [4]). Genome annotation through the MicroScope platform (15) revealed 7,012 protein-encoding genes, 72 tRNA genes, and 24 rRNA genes, with a 63.5% G+C content.

### Nucleotide sequence accession number.

This project has been deposited at DDBJ/EMBL/GenBank under the accession no. AXBU00000000.
